# Genome Sequence of *Enterobacter cancerogenus* YZ1

**DOI:** 10.1128/genomeA.00023-13

**Published:** 2013-02-28

**Authors:** Yifeng Wei, Yu Yang, Lisha Zhou, Zhangyi Liu, Xiangyan Wang, Rentao Yang, Qingqing Su, Yuping Zhou, Jiao Zhao, Jun Yang

**Affiliations:** a Key Laboratory of Bio-Inspired Smart Interfacial Science and Technology of Ministry of Education, School of Chemistry and Environment, Beihang University, Beijing, People’s Republic of China; b Shenzhen Key Laboratory of Bioenergy, BGI, Shenzhen, People’s Republic of China

## Abstract

*Enterobacter cancerogenus* is usually known as an opportunistic human pathogen. Recently, it has attracted great attention for its capability to produce bioemulsifier, degrade xenobiotics, and resist alkalis and antibiotics. Here we report the complete genome of *Enterobacter cancerogenus* YZ1, isolated from a bran-feeding *Coleoptera* insect’s frass.

## GENOME ANNOUNCEMENT

*Enterobacter cancerogenus* (formerly named *Enterobacter taylorae*) is a facultatively anaerobic Gram-negative bacterium, which is made up of a motile and rod-shaped cell and also possesses peritrichous flagella. This bacterium is usually considered a nosocomial opportunistic pathogen that can cause infections of the bones and joints associated with severe trauma or crush injuries (1, 2). In addition, it has been shown that *E. cancerogenus* bacteria possess a high level of resistance to alkalis (3) and amoxicillin antibiotics (4) and are capable of producing bioemulsifiers (5) and degrading azo dye pollutants (6) and *L-*phosphiothricin herbicides (7). Here, we report the full genome sequence of *E. cancerogenus* YZ1, which was isolated from a *Coleoptera* insect’s frass.

The genomic DNA of *E. cancerogenus* YZ1 was extracted by using a conventional proteinase K treatment and phenol-chloroform extraction. Genomic libraries containing 0.5-, 2-, and 6-kb inserts were constructed, and 1,125 M high-quality base pairs were generated at about 225-fold coverage of the genome by using Illumina Hiseq 2000 at BGI-Shenzhen, China. The pair-end reads were assembled into 11 contigs (>10 kb in size) in 11 scaffolds using SOAPdenovo v.1.05 (8). Gene prediction was determined by using Glimmer 3.0 (9). The presence of tRNA and rRNA genes was determined through tRNAscan-SE and RNAmmer, respectively (10, 11). The G+C (mole percent) contents were calculated according to the genome sequences.

The draft *E. cancerogenus* YZ1 genome sequence contains a single circular chromosome of 4,808,813 bp with an overall GC content of 55.54%. The genes were blasted against the COG (Clusters of Orthologous Groups), NR, and KEGG databases. A total of 4,495 genes were classified into 22 COG categories. There were 82 tRNA genes with a total length of 6,433 bp, and 25 rRNA loci were found.

The metabolic networks were found according to KEGG analysis. A total of 168 genes are involved in the pathway of biodegradation and metabolisms of xenobiotics, which include polycyclic aromatic hydrocarbon, toluene, nitrotoluene, chloroalkane, herbicide of atrazine, bisphenyl, naphthalene, caprolactone, xylene, styrene, ethybenzene, and herbicide of 1,1,1-trichloro-2,2-bis(4-chlorophenyl) ethane (DDT). Analysis of the genome of *E. cancerogenus* YZ1 will help us to identify diverse biodegradation genes and, further, to elucidate the microbial degradation pathways for azo dye and other pollutants.

### Nucleotide sequence accession number. 

These whole-genome sequencing projects have been deposited at DDBJ/EMBL/GenBank under the accession number ANIC00000000. The version described in this paper is the first version.
